# Neuron–astrocyte associative memory

**DOI:** 10.1073/pnas.2417788122

**Published:** 2025-05-23

**Authors:** Leo Kozachkov, Jean-Jacques Slotine, Dmitry Krotov

**Affiliations:** ^a^Department of Brain and Cognitive Sciences, Massachusetts Institute of Technology, Cambridge, MA 02139; ^b^Thomas J. Watson Research Center, International Business Machines Research, Yorktown Heights, NY 10598; ^c^Department of Mechanical Engineering, Massachusetts Institute of Technology, Cambridge, MA 02139; ^d^Massachusetts Institute of Technology-International Business Machines, Watson Artificial Intelligence Laboratory, International Business Machines Research, Cambridge, MA 02142

**Keywords:** neuron–astrocyte network, associative memory, dynamical system

## Abstract

Astrocytes, the most abundant type of glial cell, play a fundamental role in memory. Despite most hippocampal synapses being contacted by an astrocyte, there are no current theories that explain how neurons, synapses, and astrocytes might collectively contribute to memory function. We demonstrate that fundamental aspects of astrocyte morphology and physiology naturally lead to a dynamic, high-capacity associative memory system. The neuron–astrocyte networks generated by our framework are closely related to popular machine learning architectures known as Dense Associative Memories. Adjusting the connectivity pattern, the model developed here leads to a family of associative memory networks that includes a Dense Associative Memory and a Transformer as two limiting cases. In the known biological implementations of Dense Associative Memories, the ratio of stored memories to the number of neurons remains constant, despite the growth of the network size. Our work demonstrates that neuron–astrocyte networks follow a superior memory scaling law, outperforming known biological implementations of Dense Associative Memory. Our model suggests an exciting and previously unnoticed possibility that memories could be stored, at least in part, within the network of astrocyte processes rather than solely in the synaptic weights between neurons.

## Significance

Recent experiments have challenged the belief that glial cells, which compose at least half of brain cells, are just passive support structures. Despite this, a clear understanding of how neurons and glia work together for brain function is missing. To close this gap, we present a theory of neuron–astrocytes networks for memory processing, using the Dense Associative Memory framework. Our findings suggest that astrocytes can serve as natural units for implementing this network in biological “hardware.” Astrocytes enhance the memory capacity of the network. This boost originates from storing memories in the network of astrocytic processes, not just in synapses, as commonly believed. These process-to-process communications likely occur in the brain and could help explain its impressive memory processing capabilities.

Not all brain cells are neurons. It is estimated that about half of the cells in the human brain are glial cells (from “glue” in Greek) (1). Glial cells have long been known to play an important role in homeostatic brain functions, such as regulating blood flow (2)—thus contributing to hemodynamic signals such as those measured in fMRI (3)—and removing synaptic debris. Converging lines of recent evidence strongly suggest that they are also directly involved in learning, memory, and cognition (4–10). Among glial cells, astrocytes are particularly important for brain function. They serve a crucial role in directly sensing neural activity and, in turn, regulating synaptic strength and plasticity (4, 5, 11–14). In addition to sensing neural activity, astrocytes are also important targets of neuromodulatory signals such as norepinephrine and acetylcholine emerging from potentially distant brain structures such as the brainstem (15).

Of particular relevance to the computational neuroscience community are the recent findings that 1) astrocytes are necessary for forming and retrieving long-term memories (i.e., by participating in engrams) (6, 16–20) and 2) astrocytes respond to neural activity on timescales spanning many orders of magnitude, from several hundred milliseconds to minutes (14, 21, 22). Despite extensive evidence establishing the importance of neuron–astrocyte interactions for long-term memory function, computational theories of these interactions are still in their infancy.

### 1.1. What Shapes Astrocytic Computation?

The core proposal of this paper is that astrocytes compute, and these computations are shaped by tunable signaling pathways within astrocytes. We will be primarily concerned with associative computations: How neurons, synapses, and astrocytes work together to store and retrieve memories. In this case, astrocytic ${\text{Ca}}^{2+}$ flux coefficients are the site of memory storage, and neuron–synapse–astrocyte interactions are the mechanism of memory retrieval. This proposal harmoniously extends decades of prior work suggesting that memories are stored in synaptic weights (23, 24) and provides a perspective where synaptic weights “emerge” from interactions between neurons and astrocytes. Note that our proposal remains consistent with the engram hypothesis, in emphasizing that neural activation during a learning event is essential for subsequent memory recall (25, 26). Indeed, our work closely aligns with recent experimental findings indicating that astrocytes collaborate with neurons to store and retrieve memories through engram representations (20).

## 2. Neuron–Astrocyte Model

**Fig. 1.**
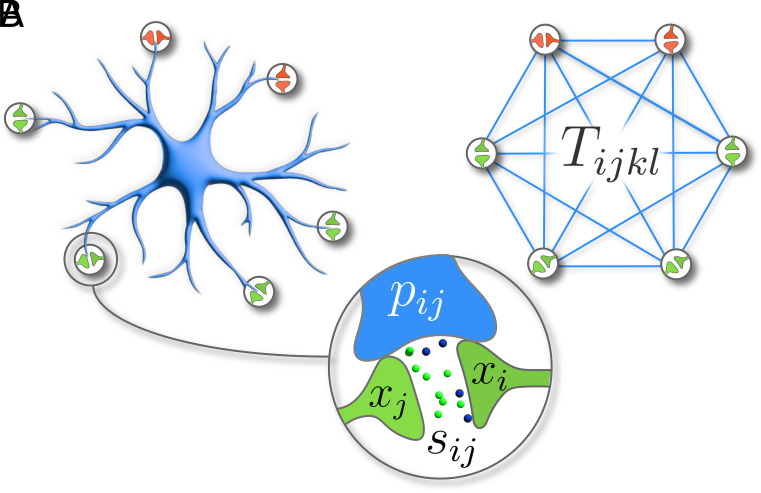
(*A*) An abstracted version of an astrocyte, showing the astrocyte processes and the synapses. (*B*) Our mathematical idealization of the minicircuit defined by a single astrocyte.

Astrocytes have a primary cell body (soma) with numerous branching processes that envelope nearby synapses (Fig. 1). This three-part structure is known as the tripartite synapse (27). A single astrocyte can form over $10^{6}$ tripartite synapses (28), and astrocyte networks spatially tile the brain, forming nonoverlapping “islands” (29). Astrocyte processes detect neurotransmitters in the synaptic cleft, leading to an upsurge in intracellular free calcium ${\text{Ca}}^{2+}$ions within the astrocyte process. This leads to a biochemical cascade in the astrocyte, potentially culminating in the release of gliotransmitters back into the synaptic cleft, influencing neural activity—a closed feedback loop. Astrocyte processes can intercommunicate through calcium transport (30), and individual astrocytes connect via gap junctions. The interplay between neurons and astrocytes, spanning multiple temporal and spatial scales, underscores the relevance of astrocytes in learning and memory. For this paper, we will focus on the following salient aspects of astrocyte biology:

- A single astrocyte can connect to millions of nearby synapses, forming three-part connections (astrocyte process, presynaptic neuron, postsynaptic neuron) called tripartite synapses (27).
- Astrocytes detect neural activity and respond by regulating this activity through the release of gliotransmitters (31).
- Tripartite synapses can interact with each other, possibly through astrocytic intracellular calcium transport (30).

### 2.1. Neural Dynamics.

The above points may be formalized into a set of dynamical equations governing the time evolution of neurons, synapses, and astrocytes. The membrane voltage $x_{i}$ for each neuron i evolves according to a standard rate recurrent neural network model (32, 33) with the characteristic time scale of the neural dynamics $\tau_{n}$, and the leak rate λ

[1] $$\tau_{n}\;{\dot{x}}_{i}=-\lambda \;x_{i}\;+\;\sum_{j=1}^{N}\;g\left(s_{\mathrm{ij}}\right)\;\phi \left(x_{j}\right)+b_{i}$$

Each neuron has an input $b_{i}$, which establishes the neuron’s baseline activation. The nonlinearity $\phi (x_{j})$ transforms neural membrane voltages into firing rates, while the term $g(s_{\mathrm{ij}})$ indicates the strength of the synaptic weight connecting neurons i and j. The variable $s_{\mathrm{ij}}$ is dynamic and alters depending on the activity of both neurons and astrocytes, as we will detail next. Note that for fixed $s_{\mathrm{ij}}$, this model is simply a standard recurrent neural network.

Box 1: **Examples of possible Lagrangian fuctions. Here, the variable z is an arbitrary dynamical variable in our model (e.g., astrocyte calcium level). Recall from the main text that activation functions are defined from the Lagrangians as** $\frac{\partial L}{\partial z_{i}}$. **The first Lagrangian provides an example of a “collective” activation function. The second Lagrangian leads to an element-wise activation function, assuming** $\frac{\partial Q}{\partial z_{i}}=q\left(z_{i}\right)$. **Generally, the only mathematical requirement for our Lagrangians is that they must be convex functions.**

Examples of Possible Lagrangians and Activations

[2] $$\begin{array}{cc} & L\left(z\right)=\;\log\sum_{i=1}^{N}e^{z_{i}}\;\to \;\;\frac{\partial L(z)}{\partial z}=\;\text{Softmax}\left(z\right)\\ & L\left(z\right)=\sum_{i=1}^{N}Q\left(z_{i}\right)\;\;\to \;\;\frac{\partial L(z)}{\partial z}=[q\left(z_{1}\right),\cdots ,q\left(z_{n}\right){]}^{⊤} \end{array}$$

### 2.2. Synaptic Dynamics.

The level of synaptic facilitation, which is denoted by $s_{\mathrm{ij}}$, refers to the degree to which presynaptic spiking activity impacts the postsynaptic neuron. Biophysically, there are many factors which influence the efficacy of a synapse, such as the number of postsynaptic receptors and the level of calcium in the synaptic cleft. The strength of the synapse can either increase or decrease based on both pre- and postsynaptic activity, as observed in Hebbian plasticity. As in earlier studies of neuron–glial interactions, we consider tripartite synapses—synapses whose plasticity is modulated by an enveloping astrocytic process, $p_{\mathrm{ij}}$

[3] $$\tau_{s}\;{\dot{s}}_{\mathrm{ij}}=-\alpha \;s_{\mathrm{ij}}+f\left(x_{i},x_{j},p_{\mathrm{ij}},s_{\mathrm{ij}}\right)+c_{\mathrm{ij}}$$

The timescale of the synaptic dynamics is $\tau_{s}$, and α establishes the leak-rate of synaptic facilitation. The function f encapsulates the interactions between these three biological variables. The inputs $c_{\mathrm{ij}}$ serve as bias variables, controlling the baseline rate of synaptic facilitation in the absence of external input. The concentration of intracellular ${\text{Ca}}^{2+}$ions in the astrocytic process that wraps around the particular synapse i−j is denoted by $p_{\mathrm{ij}}$. Biophysically, astrocytes influence neural activity through ${\text{Ca}}^{2+}$-dependent exocytosis of gliotransmitters such as GABA, D-serine, ATP, and glutamate (14)—an influence which is encoded in the function f. The level of intracellular astrocytic ${\text{Ca}}^{2+}$ is a dynamic variable and its value depends on both the calcium levels in adjacent astrocyte processes and the synaptic state $s_{\mathrm{ij}}$.

### 2.3. Astrocyte Process Dynamics.

The state of a specific astrocytic process is determined by its interactions with neurons at the tripartite synapse, plus its interactions with other processes through intracellular calcium transport

[4] $$\tau_{p}\;{\dot{p}}_{\mathrm{ij}}=-\gamma \;p_{\mathrm{ij}}\;+\;\sum_{k,l=1}^{N}\;T_{\mathrm{ijkl}}\;\psi \left(p_{\mathrm{kl}}\right)+\kappa \left(s_{\mathrm{ij}}\right)+d_{\mathrm{ij}}$$

The double sum in the astrocyte equations captures the interactions between process $p_{\mathrm{ij}}$ and all other processes (34). In the simplest scenario, calcium can diffuse between processes, resulting in a linear function ψ and tensor $T_{\mathrm{ijkl}}$ describing concentration fluxes. More complex calcium transport mechanisms between different processes within an astrocyte can produce nonlinear functions ψ. The term $d_{\mathrm{ij}}$ is a constant bias term which sets the overall “tone” of the astrocyte. This variable may be thought of as a neuromodulatory signal, potentially arriving from distant brain regions such as the pons. The input from process $p_{\mathrm{kl}}$ to process $p_{\mathrm{ij}}$ is weighed by the scalar $T_{\mathrm{ijkl}}$. A zero value for this scalar indicates no direct physical connection between process ij and kl. Hence, the astrocyte’s anatomical structure can be encoded in the nonzero entries of tensor T. The nonlinear function κ encapsulates the synapse → astrocyte signaling pathway at the tripartite synapse level. Here, $\tau_{p}$ represents the astrocyte timescale, while γ>0 is a leak term for the intracellular calcium in the astrocyte process.

## 3. Associative Neuron–Astrocyte Model

In Section 2, we described a general framework, grounded in the biology of neuron–astrocyte communication, for modeling neuron–astrocyte interactions via the tripartite synapses. Depending on the choices of the nonlinearities and parameters this network can exhibit many sophisticated dynamical behaviors—such as chaos or limit cycles—which can be difficult to analyze in full generality.

To better understand the potential role of neuron–astrocyte interactions, we will focus on an important limiting case where the system demonstrates associative memory functions. As in essentially all models of biological associative memory, this requires the presence of symmetries in the governing equations of the biological circuit. We show that under certain conditions, the resulting neuron–astrocyte model has a global energy function (Lyapunov function), which monotonically decreases on the dynamical trajectory and is bounded from below. This makes it possible to identify a regime of operation of the neuron–astrocyte network that results in dynamical trajectories converging to fixed point attractor states. The fixed points can be identified with “memories” stored in the weight matrices, and the entire neuron–astrocyte model can be regarded as an energy-based Dense Associative Memory (also known as Modern Hopfield Network) (35, 36). Importantly, this framework allows us to show that the presence of a single astrocyte can provably boost the memory capacity per compute unit of a neural circuit by a factor of N.

We will follow the general formulation of energy-based associative memories (37–39), which starts with picking three Lagrangians, which define layers of our architecture (neurons, synapses, and astrocytic processes), and the corresponding activation functions. These Lagrangians are a neural Lagrangian $L^{\left[n\right]}$, a synaptic Lagrangian $L^{\left[s\right]}$, and an astrocyte process Lagrangian $L^{\left[p\right]}$. In general these scalar functions can be arbitrary (differentiable) functions of the corresponding dynamical variables. The details of our derivation can be found in *SI Appendix*, section 1.

From these Lagrangians, we can derive via a Legendre transformation three terms in the overall energy function of the neuron–astrocyte system: $E^{\left[n\right]}$, $E^{\left[s\right]}$, and $E^{\left[p\right]}$. The activation functions in our model are dictated by the Lagrangians—indeed, the $i^{\text{th}}$ activation is simply the partial derivative of the Lagrangian with respect to the $i^{\text{th}}$ dynamical variable (*SI Appendix*, section 1 and Box 1). The remaining contributions to the total energy of the system describe the interactions between neurons, synapses, and astrocytes. These contributions describe the synapse-mediated interactions between the neurons $E^{\left[ns\right]}$, the interactions between the processes and the synapses $E^{\left[ps\right]}$, and the interactions between the individual processes inside the astrocyte $E^{\left[pp\right]}$.

The overall energy function of the neuron–astrocyte model can now be written as the sum of these six terms

[5] $$E=E^{\left[n\right]}+E^{\left[s\right]}+E^{\left[p\right]}+E^{\left[ns\right]}+E^{\left[ps\right]}+E^{\left[pp\right]}$$

From this Lagrangian formalism (37–39), the dynamical equations for the associative neuron–astrocyte energy can be viewed as the negative gradient (with respect to the nonlinearities):

[6] $$\begin{array}{c} \left\{\begin{array}{c} \begin{array}{cc} \tau_{n}\;{\dot{x}}_{i} & =-\frac{\partial E}{\partial \phi_{i}}=-\;\lambda \;x_{i}+\sum_{j=1}^{N}g_{\mathrm{ij}}\phi_{j}\;\\ \tau_{s}\;{\dot{s}}_{\mathrm{ij}} & =-2\frac{\partial E}{\partial g_{\mathrm{ij}}}=-\;\alpha \;s_{\mathrm{ij}}+\phi_{i}\phi_{j}\;+\;\psi_{\mathrm{ij}}\;\\ \tau_{p}\;{\dot{p}}_{\mathrm{ij}} & =-2\frac{\partial E}{\partial \psi_{\mathrm{ij}}}=-\;\gamma \;p_{\mathrm{ij}}+\sum_{k,l=1}^{N}T_{\mathrm{ijkl}}\;\psi_{\mathrm{kl}}\;+\;g_{\mathrm{ij}}\; \end{array} \end{array}\right. \end{array}$$

The energy-based equations have a large amount of symmetry—both in the parameters and the dynamical degrees of freedom, e.g., $T_{\mathrm{ijkl}}=T_{\mathrm{klij}}$, see *SI Appendix*, section 1. These symmetries are needed for the existence of the global energy function for our neuron–astrocyte network, which leads to mathematical tractability. In real biology, some (or all) of these symmetries might be broken, and the analytical tractability might be more difficult or even impossible. We use the energy-based model to establish theoretically the memory storage capabilities of our model. The nonsymmetric model is studied numerically in Section 4 where we show that it possesses similar capabilities despite lacking the energy-based formulation. Note that unlike the symmetries in the original Hopfield networks (40, 41) (which have no known biological interpretation), the invariance of T with respect to swapping indices ij and kl can be viewed as a natural consequence of the underlying symmetry of calcium diffusion.

The first two equations in Eq. **6** are reminiscent of the approach by Dong and Hopfield (42), which describes both the neural dynamics and synaptic plasticity by a single energy function. The difference of our system compared to ref. 42 is the existence of the network of astrocytic processes, which interact with each other and with the synapses. Following the general Lagrangian formalism, it can be shown (*SI Appendix*, section 2) that

[7] $$\begin{array}{cc} \frac{\mathrm{dE}}{\mathrm{dt}} & =-[\tau_{n}\sum_{i,j=1}^{N}{\dot{x}}_{i}\;\frac{\partial^{2}L^{\left[n\right]}}{\partial x_{i}\partial x_{j}}\;{\dot{x}}_{j}\;\\ & \;+\;\;\frac{\tau_{s}}{2}\sum_{i,j,k,l=1}^{N}{\dot{s}}_{\mathrm{ij}}\;\frac{\partial^{2}L^{\left[s\right]}}{\partial s_{\mathrm{ij}}\partial s_{\mathrm{kl}}}\;{\dot{s}}_{\mathrm{kl}}\;\\ & \;+\;\frac{\tau_{p}}{2}\sum_{i,j,k,l=1}^{N}{\dot{p}}_{\mathrm{ij}}\;\frac{\partial^{2}L^{\left[p\right]}}{\partial p_{\mathrm{ij}}\partial p_{\mathrm{kl}}}\;{\dot{p}}_{\mathrm{kl}}\;]\\ & \leq \;0 \end{array}$$

The last inequality sign holds if each Lagrangian has a positive semidefinite Hessian matrix. When the Hessian matrices are strictly positive definite, the dynamical Eq. **6** are guaranteed to arrive at a fixed point, because the energy is bounded from below [through the invariant set theorem (43)]. Thus, starting from an initial state the network dynamics flows toward one of the fixed points and for this reason describes the operation of an associative memory.

### 3.1. Connection to Dense Associative Memory.

Energy-based neuron–astrocyte networks are described by a sophisticated system of nonlinear differential equations [**6**] that are guaranteed to represent dynamical trajectories converging to fixed points attractors, assuming certain conditions on the Lagrangians are met. The locations of those fixed points $x_{i}^{*}$, $s_{\mathrm{ij}}^{*}$, and $p_{\mathrm{ij}}^{*}$ coincide with the local minima of the energy function Eq. **5**, and are independent of the time scales $\tau_{n}$, $\tau_{s}$, and $\tau_{p}$. The kinetics of the model (i.e., the shape of dynamical trajectories), however, heavily depends on these time scales. Although the characteristic time scales of synaptic plasticity and dynamics of processes (as well as the dynamics of the entire astrocyte) are subjects of active debates in the community, it is generally believed that neurons operate on faster time scales than synaptic plasticity or the processes, $\tau_{n}\ll \tau_{s},\tau_{p}$. Since the goal of this section is to analyze the fixed points of this network, which are independent of these time scales, we have a freedom to choose the kinetic time scales in a way dictated by mathematical convenience, rather than biological reality. Specifically, we will derive an effective dynamics on neurons that arises after the synapses and astrocytes are integrated out from the dynamical equations, which intuitively^*^ is possible if $\tau_{s},\tau_{p}\ll \tau_{n}$. Despite this “unbiological” choice for the intermediate steps, the final answer represents accurate locations of fixed points for the network that operates in the “biological” regime $\tau_{n}\ll \tau_{s},\tau_{p}$, or with any other choice of time scales.

The fixed points of the synaptic and processes’ dynamics in Eq. **6** are defined by (assuming for simplicity that α=γ=0)

[8] $$\begin{array}{c} \left\{\begin{array}{c} \psi_{\mathrm{ij}}=-\phi_{i}\phi_{j}\\ g_{\mathrm{ij}}=\sum_{k,l=1}^{N}T_{\mathrm{ijkl}}\phi_{k}\phi_{l} \end{array}\right. \end{array}$$

Note that for a fixed value of $\phi_{i}$, these equations uniquely determine the values of $s_{\mathrm{ij}}$ and $p_{\mathrm{ij}}$ when g and ψ are strictly monotonic. Substituting this solution into the first Eq. **6**, gives the effective dynamics for neurons (assuming for simplicity that λ=1)

[9] $$\tau_{n}{\dot{x}}_{i}=-x_{i}+\sum_{j,k,l=1}^{N}T_{\mathrm{ijkl}}\;\phi_{j}\phi_{k}\phi_{l}$$

and effective energy

[10] $$E^{\text{eff}}=[\sum_{i=1}^{N}x_{i}\phi_{i}-\;L^{\left[n\right]}]-\frac{1}{4}\sum_{i,j,k,l=1}^{N}T_{\mathrm{ijkl}}\;\phi_{i}\phi_{j}\phi_{k}\phi_{l}$$

Eqs. **9** and **10** contain the essence of our theoretical argument. The fixed points of this effective dynamical system exactly coincide with the fixed points of the original complete energy-based neuron–astrocyte network [**6**], projected on the neuron-only subspace. The hallmark of this effective theory is the existence of the four-body neuron-to-neuron interactions, represented by the product of four firing rate functions $\phi_{i}$ in the effective Lyapunov function and the product of three firing rate functions in the effective equations. In conventional firing rate models there is only one firing rate function in the right hand side of the dynamical equations and two firing rate functions in the corresponding energy function, see for example ref. 33. This is a mathematical reflection of the biological fact that each synapse connects two neurons (pre- and postsynaptic cells). In our model, we have demonstrated that the contribution of the astrocyte is to effectively create a computational many-neuron synapse (mediated by the network of astrocytic processes). In other words, the computational function of the astrocyte is to bring the information about the states of distant synapses (and neurons) to each tripartite synapse resulting in the “effective” four-neuron synapse that connects neurons that are potentially very far away from each other. In what follows, we will explain that this computational property has important implications for storing memories.

### 3.2. Storing Memories in the Network of Astrocyte Processes.

Imagine that we are given K memory patterns $\xi^{\mu }$ (index μ=1...K), and each pattern is an N-dimensional vector. The task of associative memory is to store these patterns in the weights of the neural-astrocyte network, so that temporal dynamics can asymptotically flow to these patterns. Choose tensor T such that it satisfies the following relationship

[11] $$T_{\mathrm{ijkl}}\equiv \sum_{\mu =1}^{K}\xi_{i}^{\mu }\;\xi_{j}^{\mu }\;\xi_{k}^{\mu }\;\xi_{l}^{\mu }$$

With these notations, the effective neuron-only theory is equivalent to a model with quartic interaction from the Dense Associative Memory family (35, 36). Specifically, the effective energy can be written as

$$\begin{array}{cc} E^{\text{eff}}=[\sum_{i=1}^{N}x_{i}\phi_{i}-\;L^{\left[n\right]}] & -\sum_{\mu =1}^{K}F(\sum_{i=1}^{N}\xi_{i}^{\mu }\phi_{i}),\\ & \mathrm{where}\;F\left(z\right)=\frac{1}{4}z^{4} \end{array}$$

and effective equations as

[12] $$\tau_{n}{\dot{x}}_{i}=-x_{i}+\sum_{\mu =1}^{K}\xi_{i}^{\mu }F^{\prime }(\sum_{j=1}^{N}\xi_{j}^{\mu }\phi_{j})$$

Dense Associative Memories is a new class of models that extend traditional Hopfield Networks (33, 40, 41) by introducing higher than quadratic terms in their energy function. It has been shown that this extension leads to superior information storage capacity and representational power of these models compared to the traditional Hopfield Networks (35). They are also related to the attention mechanism in Transformers (37, 45) and are used in state-of-the-art, energy-based neural networks (46, 47).

#### 3.2.1. Memory capacity of a neuron–astrocyte network.

It is insightful to ask the question: How many memories can the model store per the number of compute units? Assuming a conservative definition^†^ of the “compute unit,” our energy-based neuron–astrocyte model [**6**] has approximately $N^{2}$ compute units in the limit of large N, i.e.

$$N\;\;\text{neurons}+N^{2}\;\;\text{synapses}+N^{2}\;\;\text{processes}∼N^{2}\;\;\text{compute units}$$

The storage capacity $K^{\text{max}}$ of the Dense Associative Memory model with quartic energy is known to be (35)

$$K^{\text{max}}\;∼\;N^{3}$$

Thus, for our model the number of memories per compute unit grows linearly as the size of the network is increased

$$\frac{K^{\text{max}}}{\;\text{Number of compute units}}\;∼\;N$$

This metric can be compared with other biologically plausible implementations of Dense Associative Memory. For instance, Krotov and Hopfield (37) have proposed to augment the network of feature neurons with a set of auxiliary hidden neurons. In their model, the compute units consist of both feature and hidden neurons, and importantly the number of memories per compute unit is a constant independent of N,

$$\frac{K^{\text{max}}}{\;\text{Number of compute units}}\;∼\;constant$$

**Fig. 2.**
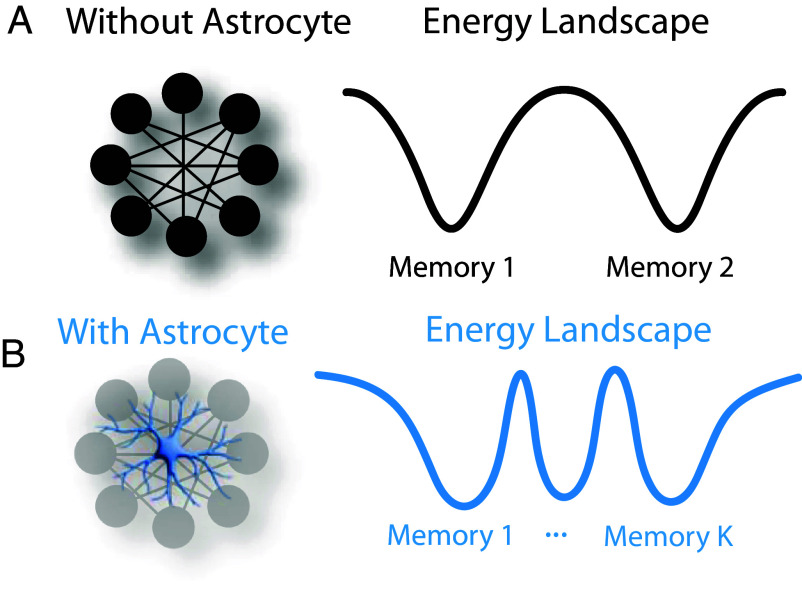
For a fixed number of neurons, the neuron–astrocyte network is capable of storing many more memories than the neuron-only network. (*A*) The energy landscape of a neuron-only associative network. (*B*) The energy landscape of a neuron–astrocyte associative network. The memories are more densely packed into the state-space, thereby enabling superior memory storage and retrieval capabilities.

Thus, neuron–astrocyte networks significantly outperform the implementation (37) according to this metric, see Fig. 2. This makes neuron–astrocyte networks an exciting candidate for biological “hardware” implementing Dense Associative Memory.

Even more insightful is to trace down the origin of this memory storage back to the biophysical implementation of the neuron–astrocyte networks Eq. **6**. The memories in our model are stored in tensor T, which describes the network of astrocyte’s processes and the transport of ${\text{Ca}}^{2+}$ or other potential molecules, e.g. protein kinase A, between those processes. Our theory suggests that memories can be stored in the biological machinery (inside a single astrocyte) of transport of an appropriate signaling molecule between the astrocyte’s processes. We have demonstrated a conceptual theoretical possibility of such a storage through a Hebbian-like plasticity rule [**11**]. This was done for the sake of transparency of the theoretical argument. In principle, more sophisticated storage rules are possible too. We hope that future experiments might be able to test this exciting hypothesis.

#### 3.2.2. How many astrocyte parameters are needed?.

The Hebbian-like storage scheme Eq. **11** requires that every process within the astrocyte be directly connected to every other process. Future experiments will hopefully determine whether such detailed communication within a single astrocyte is possible. In the meantime, it is instructive to ask how the memory capacity changes as a function of the degree of process-to-process connectivity.

Heuristically, if we wish to store K memories, each containing N independent bits, we need on the order of KN parameters in our model—one parameter for each bit of information stored. For example, if we wish to store K=N memories, we need on the order of $N^{2}$ parameters—these parameters can be stored inside the N×N weight matrix of the traditional Hopfield network. For our neuron–astrocyte network, the number of parameters can be written as $rN^{2}$, where r is the number of connections per astrocyte process. For an all-to-all network as we have described above, $r=N^{2}$. Thus, the number of connections per process needed to store K memories is given by

$$KN=rN^{2}\;\Rightarrow \;r=\frac{K}{N}$$

This equation tells us that if we wish to achieve linear storage capacity (i.e., K=N), then we can ignore process-to-process connectivity, since r=const means that each astrocyte process is an isolated dynamical variable. If we wish to achieve supralinear memory storage, one way to do so is by connecting the astrocyte processes to one another. Biologically, parameter r can be larger than constant, but smaller than $N^{2}$. Thus, this equation provides the number of memories that can be stored given a particular value of r – a quantity which can in principle be determined experimentally.

#### 3.2.3. Connection to transformers models.

It is worth noting that having detailed entries of the tensor $T_{\mathrm{ijkl}}$ is not necessary for our neuron–astrocyte model to perform interesting computations. Indeed, one can demonstrate (*SI Appendix*, section 3) that setting $T_{\mathrm{ijkl}}=1$ in Eq. **4** results in a stable dynamical model whose equilibrium states approximate the output of a transformer’s self-attention mechanism (48). This connection also implies that our neuron–astrocyte model can be made to smoothly transition between a transformer on the one hand and a Dense Associative Memory network on the other.

## 4. Simulations

In this section, we conduct two computational experiments. The first uses the energy-based Eq. **6** with the Hebbian-like learning rule Eq. **11**. The second employs backpropagation-through-time, foregoing symmetry requirements. The aim of the first experiment is to validate our theoretical claims. The second experiment aims to demonstrate that strong symmetry is not necessary, but rather sufficient, for the system to exhibit associative memory function. This point is crucial in the context of biology, considering the difficulty of achieving “pure” symmetry on noisy biological hardware. The code to reproduce these experiments is available at this GitHub repository.

### 4.1. Energy-Based Experiments.

To demonstrate that the memory storage scheme Eq. **11** above works in practice, we performed numerical experiments using the CIFAR10 dataset. *SI Appendix*, Fig. S2 shows the result of retrieving four different memories after encoding K=25 memories in the network. As predicted by theory, in each case, the neuron–astrocyte network converges to fixed points with the correct neural attractor (i.e., the attractor corresponding to the stored memory). Along trajectories of the neuron–astrocyte network, the energy function is monotonically decreasing. The details of the training are given in *SI Appendix*, section 4. Additionally, we have conducted energy-based experiments on audio data, which can be found in *SI Appendix*, section 5.

### 4.2. Backpropagation-Based Experiments.

**Fig. 3.**
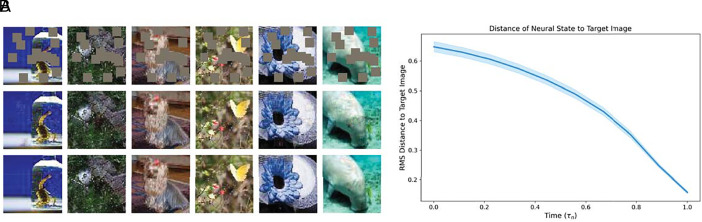
(*A*) Error-correcting capabilities of the neuron–astrocyte network, trained with backpropagation, demonstrated with images from the Tiny ImageNet dataset (49). *Top* row is the masked input to the network, *Middle* row is the final state of our network, *Bottom* row is the ground-truth, unmasked image. (*B*) Root-mean-squared distance of the state of our network to the ground-truth image, as function of time. SE was calculated across a batch of 64 images.

In order to show that our network does not require large amounts of symmetry to perform associative memory functions, we trained it on a self-supervised learning task using the Tiny ImageNet dataset (49). Specifically, given a batch of ImageNet images, downsampled to 64×64, we randomly masked fifteen square patches, each ten pixels across (roughly 40% of all pixels). The exact positions of the mask patches were varied across batches. The masked images were provided to the network as an initial state, and then the state of the network was dynamically evolved for prespecified number of steps. The goal of training was to minimize the difference (as measured by mean squared error) between the network output and the unmasked images. The parameters of the network were initialized randomly and optimized using Backpropagation-Through-Time (BPTT). The results are shown in Fig. 3. More details on the training process can be found in *SI Appendix*, section 5.

Finally, we note that the energy-based model can also be trained using implicit techniques such as recurrent backpropagation (50, 51).

## 5. Discussion

We have introduced a biologically inspired model that describes the interactions between neurons, synapses, and astrocytes. In our model, astrocytes are able to adaptively control synaptic weights in an online fashion. Theoretical analysis has demonstrated that this model can exhibit associative memory behavior and is closely related to the Dense Associative Memory family of models with supralinear memory capacity, as well as to transformers. We have shown that, through the choice of the connectivity tensor $T_{\mathrm{ijkl}}$, our neuron–astrocyte model can be smoothly dialed between operating as a transformer and operating as a Dense Associative Memory network. This opens up the possibility for exploring novel architectures “in-between” transformers and Dense Associative Memories. Furthermore, we have presented a simple algorithm for memory storage and have provided numerical evidence of our models’ effectiveness, such as successfully storing and retrieving CIFAR10 and ImageNet images.

In broader terms, this work proposes that memories can, at least in part, be stored within the molecular machinery of astrocytes. This contrasts with the prevailing neuroscience viewpoint that memories are stored in the synaptic weights between neurons. To experimentally validate this claim, one would need to selectively interfere with the ability of ${\text{Ca}}^{2+}$ to diffuse intracellularly through astrocytes. Our model predicts that hindering this diffusion would significantly impair memory recall. Our model is flexible enough to accommodate many different types of process-to-process coupling patterns, which could presumably be fit to match physiological data. For example, it is possible to enforce “nearest-neighbor” coupling between astrocyte processes (which can be achieved by e.g., imposing a block-diagonal structure on the tensor T such that $T_{\mathrm{ijkl}}=0$ if processes ij and kl are not spatially close to each other), while still guaranteeing convergence of our model to a fixed point.

The feedback loop between neurons and astrocytes is well established experimentally, yet a simple computational account of its function remains largely absent. Previous models have primarily focused on the biophysical details of neuron–astrocyte interactions, aiming to capture the emergence of complex spatiotemporal calcium dynamics (52, 53). Building on these efforts, our work highlights the role of astrocytic modulation in synaptic plasticity. However, rather than modeling detailed biophysical mechanisms, we take a higher-level approach, using a “firing rate” model to abstract away these complexities and uncover the core computational principles governing neuron–astrocyte interactions.

One particularly intriguing feature of astrocytes is their abundance in the brain. Indeed, they are present in virtually every major brain structure. Of particular relevance to the modeling approach introduced here are associative brain areas, such as the neocortex and hippocampus, which are believed to play a crucial role in memory storage and retrieval. Notably, human neocortical astrocytes are significantly larger and more active than their rodent counterparts, suggesting an enhanced computational function (54). As in prior modeling work using recurrent networks (55), our neuron–astrocyte model can be tuned (i.e., trained) to perform functions associated with specific tasks relevant to particular brain regions. For example, astrocytes in the visual cortex may be specialized for storing and retrieving visual information, whereas those in language-related areas may be more attuned to processing auditory information.

While our focus has been on a minicircuit consisting of a single astrocyte interacting with multiple nearby synapses, astrocytes also extensively communicate with each other through chemical gap junctions. Exploring the implications of this intercellular coupling will be the subject of future research.

Key ideas in machine learning and AI drew initial inspiration from neuroscience, including neural networks, convolutional nets, threshold linear (ReLu) units, and dropout. Yet it is debatable whether neuroscience research from the last fifty years has significantly influenced or informed machine learning. Astrocytes, along with other biological structures such as dendrites (56) and neuromodulators (57) may offer a fresh source of inspiration for building state-of-the-art AI systems.

## Supplementary Material

Appendix 01 (PDF)

## Data Availability

Code data have been deposited in GitHub (https://github.com/kozleo/naam) (58). All other data are included in the manuscript and/or *SI Appendix*.

## Footnotes

^*^Strictly speaking, specific conditions must be satisfied in order for this operation to be well-defined and to avoid pathologies such as peaking phenomena. In our system this can easily be done by restricting the eigenvalues of T to be less than γ, which ensures that the fast dynamics are contracting (44).

^†^A less conservative definition would consider the entire astrocyte as a single compute unit.
