# Supplementary material for: Neuron–astrocyte associative memory
Source: Proc Natl Acad Sci U S A. 2025 May 23;122(21):e2417788122. doi: 10.1073/pnas.2417788122 (PMC12130835; doi:10.1073/pnas.2417788122)
Supplement: Supplementary file 1 — Appendix 01 (PDF) [file pnas.2417788122.sapp.pdf]

Supporting Information for

Supporting Information for Neuron-Astrocyte Associative Memory

Leo Kozachkov, Jean-Jacques Slotine and Dmitry Krotov

[2]To whom correspondence should be addressed. E-mail: krotov@ibm.com

This PDF file includes:

Supporting text
Figs. S1 to S4
SI References

Supporting Information Text

1. Definitions of Lagrangians and Energy

As described in the main text, the Lagrangians are: a neural Lagrangian $\mathcal{L}^{[n]}$, a synaptic Lagrangian $\mathcal{L}^{[s]}$, and an astrocyte process Lagrangian $\mathcal{L}^{[p]}$. In general these scalar functions can be arbitrary (differentiable) functions of the corresponding dynamical variables. The activation functions are defined as partial derivatives of the Lagrangians

$$\underbrace{\mathcal{L}^{[n]}(\mathbf{x}) \quad \rightarrow \quad \phi_i \equiv \frac{\partial \mathcal{L}^{[n]}}{\partial x_i}}_{\text{Neural Lagrangian}}, \qquad \underbrace{\mathcal{L}^{[s]}(\mathbf{s}) \quad \rightarrow \quad g_{ij} \equiv \frac{\partial \mathcal{L}^{[s]}}{\partial s_{ij}}}_{\text{Synaptic Lagrangian}}, \qquad \underbrace{\mathcal{L}^{[p]}(\mathbf{p}) \quad \rightarrow \quad \psi_{ij} \equiv \frac{\partial \mathcal{L}^{[p]}}{\partial p_{ij}}}_{\text{Astrocyte Process Lagrangian}} \tag{1}$$

One possible choice of these functions is additive: summing each contribution from all the individual computational elements (e.g., individual neurons), which results in activation functions that depend only on individual computational elements – for instance, $\phi(x_i) = \tanh(x_i)$. More general choices of the Lagrangians allow for "collective" activation functions, which depend on the dynamical degrees of freedom of several or all the computational elements in a given layer, for example a softmax.

From the Lagrangians Eq. (1), we may derive via a Legendre transform three terms in the overall energy function of the neuron-astrocyte system, corresponding to three layer energies,

$$E^{[n]} + E^{[s]} + E^{[p]} = \underbrace{\lambda\Big[\sum_{i=1}^{N} x_i\phi_i - \mathcal{L}^{[n]}\Big]}_{\text{Neural Energy}} + \underbrace{\frac{\alpha}{2}\Big[\sum_{i,j=1}^{N} s_{ij}g_{ij} - \mathcal{L}^{[s]}\Big]}_{\text{Synaptic Energy}} + \underbrace{\frac{\gamma}{2}\Big[\sum_{i,j=1}^{N} p_{ij}\psi_{ij} - \mathcal{L}^{[p]}\Big]}_{\text{Astrocyte Process Energy}} \tag{2}$$

where for simplicity of the presentation we dropped the input signals, $b_i = c_{ij} = d_{ij} = 0$. The remaining contributions to the total energy of the system describe the interactions between these three layers. These contributions describe the synapse-mediated interactions between the neurons $E^{[ns]}$, the interactions between the processes and the synapses $E^{[ps]}$, and the interactions between the individual processes inside the astrocyte $E^{[pp]}$,

$$E^{[ns]} + E^{[ps]} + E^{[pp]} = -\Big[\frac{1}{2}\sum_{i,j=1}^{N} g_{ij}(\mathbf{s})\phi_i(\mathbf{x})\phi_j(\mathbf{x}) \; + \; \frac{1}{2}\sum_{i,j=1}^{N} \psi_{ij}(\mathbf{p})g_{ij}(\mathbf{s}) \; + \; \frac{1}{4}\sum_{i,j,k,l=1}^{N} T_{ijkl}\;\psi_{ij}(\mathbf{p})\psi_{kl}(\mathbf{p})\Big] \tag{3}$$

The overall energy function of the neuron-synapse-astrocyte model can now be written as the sum of these six terms

$$E = E^{[n]} + E^{[s]} + E^{[p]} + E^{[ns]} + E^{[ps]} + E^{[pp]} \tag{4}$$

As mentioned previously, the energy-based equations have a large amount of symmetry–both in the parameters and the dynamical degrees of freedom. Specifically, $s_{ij} = s_{ji}$, $g_{ij} = g_{ji}$, $p_{ij} = p_{ji}$, $\psi_{ij} = \psi_{ji}$, and $T_{ijkl} = T_{klij}$, $T_{ijkl} = T_{jikl}$, $T_{ijkl} = T_{ijlk}$. These symmetries are needed for the existence of the global energy function for our neuron-astrocyte network, which leads to mathematical tractability (in analogy to neuron-only networks (2, 3)). In real biology some (or all) of these symmetries might be broken, and the analytical tractability might be more difficult or even impossible. We use the energy-based model to establish theoretically the memory storage capabilities of our model. The non-symmetric model is studied numerically in the main text, in the Simulations section.

2. Proof of Decreasing Energy Function

The overall time derivative of the energy function may be written as

$$\frac{dE}{dt} = \sum_{i=1}^{N} \frac{\partial E}{\partial x_i}\,\dot{x}_i + \sum_{i,j=1}^{N} \frac{\partial E}{\partial s_{ij}}\,\dot{s}_{ij} + \sum_{i,j=1}^{N} \frac{\partial E}{\partial p_{ij}}\;\dot{p}_{ij}$$

which may be expressed using the chain rule as

$$\begin{aligned} \frac{dE}{dt} &= \sum_{i,j=1}^{N} \frac{\partial E}{\partial \phi_i}\frac{\partial \phi_i}{\partial x_j}\,\dot{x}_j + \sum_{i,j,k,l=1}^{N} \frac{\partial E}{\partial g_{ij}}\frac{\partial g_{ij}}{\partial s_{kl}}\,\dot{s}_{kl} + \sum_{i,j,k,l=1}^{N} \frac{\partial E}{\partial \psi_{ij}}\frac{\partial \psi_{ij}}{\partial p_{kl}}\,\dot{p}_{kl} \\ &= \sum_{i,j=1}^{N} \frac{\partial E}{\partial \phi_i}\frac{\partial^2 \mathcal{L}^{[n]}}{\partial x_i \partial x_j}\,\dot{x}_j + \sum_{i,j,k,l=1}^{N} \frac{\partial E}{\partial g_{ij}}\frac{\partial^2 \mathcal{L}^{[s]}}{\partial s_{ij}\partial s_{kl}}\,\dot{s}_{kl} + \sum_{i,j,k,l=1}^{N} \frac{\partial E}{\partial \psi_{ij}}\frac{\partial^2 \mathcal{L}^{[p]}}{\partial p_{ij}\partial p_{kl}}\,\dot{p}_{kl} \end{aligned} \tag{5}$$

The second line follows from the definition of the Lagrangians Eq. (1). Plugging the dynamics defined in equations main text equations (6) into this last expression, we get the desired result, provided that the Lagrangians are all convex (i.e., have positive semi-definite Hessians)

$$\frac{dE}{dt} = -\Big[\tau_n \sum_{i,j=1}^{N} \dot{x}_i\,\frac{\partial^2 \mathcal{L}^{[n]}}{\partial x_i \partial x_j}\,\dot{x}_j \; + \; \frac{\tau_s}{2}\sum_{i,j,k,l=1}^{N} \dot{s}_{ij}\,\frac{\partial^2 \mathcal{L}^{[s]}}{\partial s_{ij}\;\partial s_{kl}}\,\dot{s}_{kl} \; + \; \frac{\tau_p}{2}\sum_{i,j,k,l=1}^{N} \dot{p}_{ij}\,\frac{\partial^2 \mathcal{L}^{[p]}}{\partial p_{ij}\partial p_{kl}}\,\dot{p}_{kl}\Big] \leq 0 \tag{6}$$

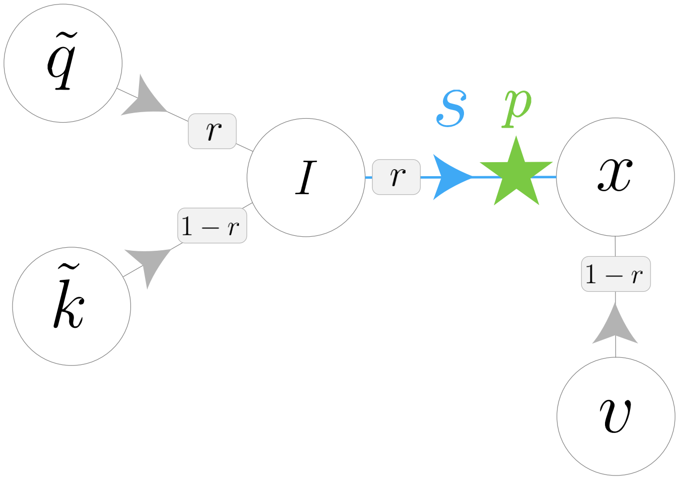

Fig. S1. Dynamic, stable neuron-astrocyte architecture which implements the self-attention operation in transformers.

3. Proof of Neuron-Astrocyte Equilibration to Transformer Output

Neuron-Astrocyte Transformer Architecture The aim of this section is to demonstrate that a simple selection of the astrocyte process-to-process weights $T_{ijkl} = 1$ is sufficient, along with a specialized architecture (S1), to produce interesting computations in the general neuron-astrocyte network equations (1), (3) and (4) in the main text. We consider a single group of N neurons, where the state of the i-th neuron in this group is denoted by x_i. These neurons receive inputs from another group of M neurons, where the state of the j-th neuron in this group is denoted I_j. The synaptic connection between neuron I_j and neuron x_i is represented by s_{ij}. The x_i neurons also receive input from another group of N neurons, whose state we denote by v_i, for reasons that will become clear later on. The dynamical equations for the x_i layer are given by

$$\tau_n \dot{x}_i = -x_i + r \sum_{j=1}^{M} s_{ij} I_j + (1-r) v_i \qquad [7]$$

where $r = \{0, 1\}$ stands for "read", and is a global parameter controlling whether the network is in "read" or "write" mode. Biologically, global coordination of this kind may be achieved by neuromodulators (e.g., acetylcholine) (1). We additionally assume that the I_j neurons receive strong input from two M-dimensional neural populations which we denote as $\tilde{q}_j$ and $\tilde{k}_j$ (again for reasons that will become clear shortly), so that the state of neuron I_j is given by

$$I_j = r\ \tilde{q}_j + (1-r)\ \tilde{k}_j \qquad [8]$$

The synaptic weights s_{ij} are modulated by an astrocyte and evolve according to the following dynamical equations:

$$\tau_s \dot{s}_{ij} = -p_{ij} s_{ij} + c_{ij} \qquad [9]$$

where p_{ij} represents the state of the astrocyte process ij, and c_{ij} is a fixed bias term. This set of synaptic equations can be associated with main text equations (3) by setting

$$\alpha = 0, \quad \text{and} \quad f(s_{ij}, x_i, x_j, p_{ij}) = -p_{ij} s_{ij}$$

The astrocyte dynamics are described by simple diffusive equations:

$$\tau_p \dot{p}_{ij} = \sum_{k=1}^{N} \sum_{l=1}^{M} [p_{kl} - p_{ij}] \quad \text{with} \quad \sum_{i=1}^{N} \sum_{j=1}^{M} p_{ij}(0) > 0 \qquad [10]$$

The inequality is to ensure that the total amount of Ca^{2+}initially in the astrocyte is positive. Biologically, even Ca^{2+}concentrations inside individual processes are positive $p_{ij}(0) \geq 0$, but, mathematically, we will only use the positivity of the total amount of calcium inside the astrocyte. Similar to the synaptic variables, this set of astrocyte equations can be associated with the main text astrocyte equations (4) by setting

$$\psi(p_{ij}) = p_{ij}, \quad \gamma = NM, \quad T_{ijkl} = 1, \quad \kappa(s_{ij}) = 0 \quad \text{and} \quad d_{ij} = 0$$

Before establishing a connection with transformer networks, we will describe the dynamical properties of Equations Eq. (7), Eq. (9), and Eq. (10). Specifically, we will demonstrate that, during the reading phase, the neurons x_i converge to an equilibrium point determined solely by the input neurons I_j, the initial Ca^{2+}concentration in the astrocyte, and the synaptic bias terms c_{ij}. Following this, we will illustrate how a judicious and biologically plausible selection of input neuron states, initial Ca^{2+}levels, and synaptic biases enables the neurons x_i to mimic the output of the self-attention mechanism in transformers.

Convergence & Synchronization of Astrocyte Processes To begin, note that the astrocyte equations Eq. (10) are autonomous with respect to the neural and synaptic variables. Therefore, we can analyze their convergence properties independently from these variables. In particular, we can show that the astrocyte equations synchronize to the average of their initial conditions. To see this, first note that the total amount of Ca^{2+}in the astrocyte, which we denoted z, is conserved throughout the diffusion process

$$z \equiv \sum_{i=1}^{N}\sum_{j=1}^{M} p_{ij} \qquad \Longrightarrow \qquad \dot{z} = \sum_{i=1}^{N}\sum_{j=1}^{M} \dot{p}_{ij} = 0$$

Second, note that this property implies that if the astrocyte processes *synchronize*, i.e., $p_{ij} = p_{kl} = p^*$, then the state of each astrocyte process must converge to the average of the astrocyte initial conditions, because

$$z(t) \;=\; NMp^* \;=\; z(0) \;=\; \sum_{i=1}^{N}\sum_{j=1}^{M} p_{ij}(0) \qquad \Longrightarrow \qquad p^* = \frac{1}{NM}\sum_{i=1}^{N}\sum_{j=1}^{M} p_{ij}(0) \;>\; 0 \tag{11}$$

The inequality follows from the assumption in Eq. (10), that the total initial amount of Ca^{2+}in the astrocyte is positive. To prove that the astrocyte processes in fact synchronize, one can use a virtual system, as in (4) or a Lyapunov-like function

$$L = \frac{1}{2}\,(p_{ij} - p_{kl})^2 \geq 0$$

for arbitrary indices ij and kl. Taking the time derivative of this function, one sees that

$$\dot{L} = (p_{ij} - p_{kl})(\dot{p}_{ij} - \dot{p}_{kl}) = -\frac{NM}{\tau_p}(p_{ij} - p_{kl})^2 = -\frac{2NM}{\tau_p}L \qquad \Longrightarrow \qquad L(t) = L(0)e^{-\frac{2NMt}{\tau_p}}$$

which shows that the astrocyte processes do in fact synchronize (i.e., $|p_{ij} - p_{kl}| \to 0$) exponentially with rate $\frac{NM}{\tau_p}$.

Convergence of Synapses Moving on to the synaptic equations Eq. (9), we will assume that the astrocyte processes have converged to $p^* > 0$. This assumption is justified because, as the preceding paragraph shows, the converge of the astrocyte process to p^* is *exponential*, meaning that p_{ij} can be brought arbitrarily close to p^* after finite time. Because c_{ij} is a constant, and because p^* is strictly positive, this implies that the synapses simply converge exponentially quickly to the value

$$s_{ij}^* = \frac{c_{ij}}{p^*} \tag{12}$$

Convergence of Neurons Following a similar logic, the neural equations Eq. (7) converge exponentially. When the network is in its writing phase (i.e., $r = 0$), the neurons converge to the equilibrium point

$$x_i^* = v_i \tag{13}$$

otherwise, when the network is in the reading phase (i.e., $r = 1$), the network converges exponentially to the equilibrium point

$$x_i^* = \sum_{j=1}^{M} s_{ij}^* I_j = \frac{1}{p^*}\sum_{j=1}^{M} c_{ij} I_j = \frac{NM \sum\limits_{j=1}^{M} c_{ij} I_j}{\sum\limits_{i=1}^{N}\sum\limits_{j=1}^{M} p_{ij}(0)} \tag{14}$$

The first equality was obtained by substituting in s_{ij}^* from Eq. (12), while the second equality was obtained by subsituting in the value of p^* from Eq. (11).

Transformer Self-Attention We are now in a position to relate the neural fixed point Eq. (14) to the output of the self-attention mechanism in transformers. To establish this connection, we define several important terms. Consider a set of K_{tok} *tokens*, which are vectors in $\mathbb{R}^D$. As is standard in transformer architectures, these tokens are transformed via three linear mappings into three new sets of vectors known as keys, queries, and values. By collecting these transformed vectors into matrices, we denote

$$K,\; Q \in \; \mathbb{R}^{K_{\text{tok}} \times D} \qquad \text{and} \qquad V \;\in\; \mathbb{R}^{K_{\text{tok}} \times N}.$$

The *self-attention* matrix A associated with these matrices is given by

$$A_{\mu i} = \sum_{\beta=1}^{K_{\text{tok}}} \frac{\exp\left(\sum\limits_{s=1}^{D} Q_{\mu s} K_{\beta s}\right) V_{\beta i}}{\sum\limits_{\sigma=1}^{K_{\text{tok}}} \exp\left(\sum\limits_{s=1}^{D} Q_{\mu s} K_{\sigma s}\right)}$$

An important characteristic of the above self-attention matrix is that it may be approximated via feature maps (5) with the following property

$$\phi(\mathbf{x})^T \phi(\mathbf{y}) \approx \exp(\mathbf{x}^T \mathbf{y})$$

where $\mathbf{x}$ and $\mathbf{y}$ are two vectors. In general, the output dimension of ϕ, which we denote M (the same M as above) is much larger than the input dimension D. To keep notations clean, we define the output of these feature maps (applied column-wise to the matrices K and Q) as

$$\tilde{K}, \tilde{Q} \;\equiv\; \phi(K), \phi(Q) \;\in\; \mathbb{R}^{K_{tok} \times M}$$

With this notation, we have that

$$A_{\mu i} \approx \sum_{\beta=1}^{K_{\text{tok}}} \frac{\sum\limits_{j=1}^{M} \tilde{Q}_{\mu j} \tilde{K}_{\beta j} V_{\beta i}}{\sum\limits_{\sigma=1}^{K_{\text{tok}}} \sum\limits_{j=1}^{M} \tilde{Q}_{\mu j} \tilde{K}_{\sigma j}}$$

Neuron-Astrocyte Self-Attention To make a connection to the fixed point equation Eq. (14), we first rearrange the above terms as follows

$$A_{\mu i} \approx \frac{\sum\limits_{j=1}^{M} \left(\sum\limits_{\beta=1}^{K_{\text{tok}}} V_{\beta i} \tilde{K}_{\beta j} \right) \tilde{Q}_{\mu j}}{\sum\limits_{j=1}^{M} \tilde{Q}_{\mu j} \sum\limits_{\sigma=1}^{K_{\text{tok}}} \tilde{K}_{\sigma j}} \qquad [15]$$

We then set the bias terms c_{ij} in the synaptic equations as follows:

$$c_{ij} = \frac{1}{M} \sum_{\beta=1}^{K_{\text{tok}}} V_{\beta i} \tilde{K}_{\beta j} \qquad [16]$$

Biologically, this corresponds to a simple form of Hebbian learning between two groups of neurons. Within the framework of Eq. (14), this can be achieved during the writing phase (i.e., $r = 0$), such that $x_i = v_i = V_{\beta i}$ and $I_j = \tilde{k}_j \equiv \tilde{K}_{\mu j}$ (from Eq. (8)). Then, updating c_{ij} by adding the product of these two terms for each β represents a simple form of associative Hebbian learning, and yields Eq. (16). Assuming c_{ij} is initially zero, we see that

$$\Delta c_{ij} = \frac{1}{M} x_i I_j = \frac{1}{M} V_{\beta i} \tilde{K}_{\beta j} \qquad \Longrightarrow \qquad c_{ij} = \frac{1}{M} \sum_{\beta=1}^{K_{\text{tok}}} V_{\beta i} \tilde{K}_{\beta j}$$

Finally, during the reading phase ($r = 1$) we select an index μ in the token sequence to run the neuron-astrocyte dynamics forward on. In other words, c_{ij} is fixed across all tokens, but I_j and $p_{ij}(0)$ change from token to token. For a particular index μ we instantiate the neurons I_j Eq. (8) and the astrocyte processes p_{ij} as follows

$$I_j = \tilde{q}_j \equiv \tilde{Q}_{\mu j} \qquad \text{and} \qquad p_{ij}(0) = \tilde{Q}_{\mu j} \sum_{\sigma=1}^{K_{tok}} \tilde{K}_{\sigma j} \qquad [17]$$

Plugging Eq. (16) and Eq. (17) into the neural fixed point condition for the reading phase Eq. (14), we arrive at the desired result

$$x_i^* = \frac{NM \sum\limits_{j=1}^{M} c_{ij} I_j}{\sum\limits_{i=1}^{N} \sum\limits_{j=1}^{M} p_{ij}(0)} = \frac{\frac{NM}{M} \sum\limits_{j=1}^{M} \sum\limits_{\beta=1}^{K_{\text{tok}}} V_{\beta i} \tilde{K}_{\beta j} \tilde{Q}_{\mu j}}{\sum\limits_{i=1}^{N} \sum\limits_{j=1}^{M} \tilde{Q}_{\mu j} \sum\limits_{\sigma=1}^{K_{\text{tok}}} \tilde{K}_{\sigma j}} = \frac{\frac{NM}{M} \sum\limits_{j=1}^{M} \left(\sum\limits_{\beta=1}^{K_{\text{tok}}} V_{\beta i} \tilde{K}_{\beta j} \right) \tilde{Q}_{\mu j}}{N \sum\limits_{j=1}^{M} \tilde{Q}_{\mu j} \sum\limits_{\sigma=1}^{K_{\text{tok}}} \tilde{K}_{\sigma j}} \approx A_{\mu i}$$

which shows that for a particular choice of parameters and initialization, the neuron-astrocyte network converges to the output of self-attention. In other words, the neural fixed point equation Eq. (14) is equal to the self-attention approximation Eq. (15).

4. Details of Energy Network Experiments

To reduce the dimensionality of the problem, we use a custom autoencoder to encode the 3072-dimensional ($32 \times 32 \times 3$) CIFAR10 images into a smaller, 768 dimensional, latent space. A single CIFAR10 image in this latent space corresponds to a single memory ξ^μ. In addition to being 768-dimensional, this latent space was also binary, so that $\xi^\mu \in [-1, 1]^{768}$. To ensure that the latent space was binary, we wrote a custom autograd function which outputs the sign of the argument during the forward pass, but is linear during the backwards pass. The discrepancy between forward and backward pass induces a small amount of gradient noise in the training process, which is not significant enough to impair learning. For concreteness, in PyTorch this custom activation is given by:

```
class RoundWithGradient(torch.autograd.Function):
    @staticmethod
    def forward(ctx, x):
        return torch.sign(x)
```

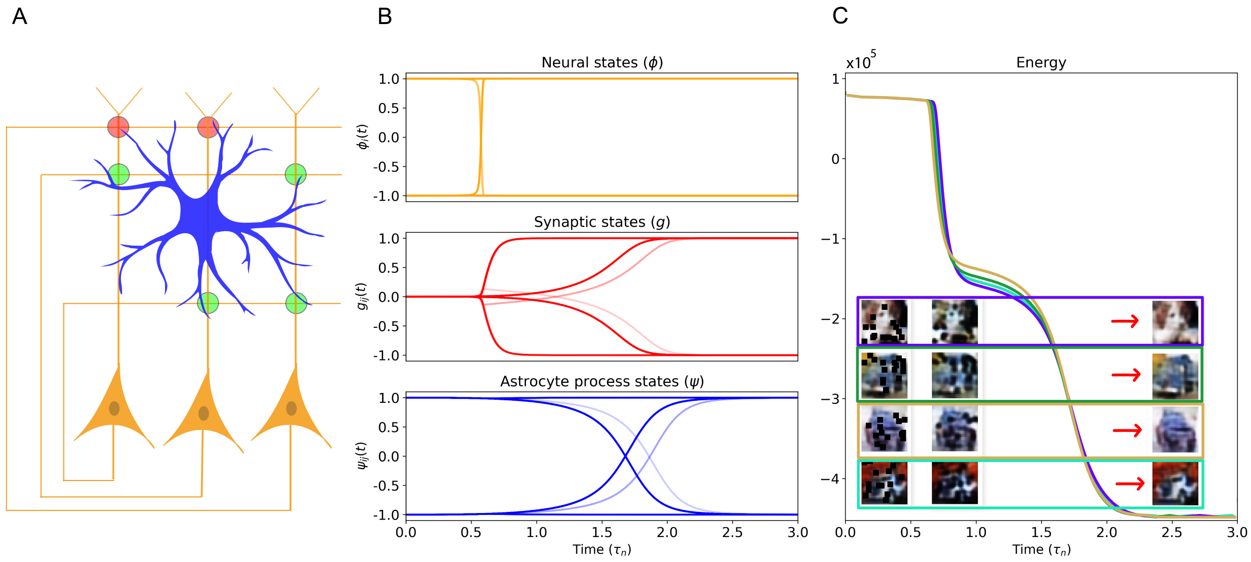

Fig. S2. A) A schematic for our associative neuron-synapse-astrocyte network. B) The neural, synaptic, and astrocyte process activations during memory retrieval. In this case, the memory item being retrieved is an image of a dog taken from the CIFAR10 dataset. C) Decreasing energy function of the neuron-synapse-astrocyte network as the dynamics evolve. The decreasing energy functions during four different retrievals are shown.

```

    @staticmethod
    def backward(ctx, grad_output):
        return grad_output


def round_with_gradient(x):
    return RoundWithGradient.apply(x)
```

To initialize the network, we reasoned (in analogy with traditional Hopfield networks) that the entire system should be initialized close to a stored memory. In our case, this includes all dynamical variables: neuron, synapses, and astrocytes. To do this, we set the time derivatives in main text equations (6) equal to zero, clamped the neural state at the corrupted memory x_0, and then solved the resulting set of algebraic equations for $p_{ij}(0)$ and $s_{ij}(0)$. Note that the synaptic states and process states are uniquely determined given a fixed neural state, due to the invertibility of g and ψ.

5. Details of Backpropagation Experiment

To reduce the dimensionality of the problem, we assume that the the state of the processes does not depend on index i, in other words $p_{ij} = p_j$. Biologically, this has the interpretation that the astrocyte processes associated with post-synaptic neuron i are all synchronized. This can be justified by assuming that nearby astrocyte processes are sensitive to inputs arrive at the dendritic tree of neuron i, and can rapidly redistribute their Ca^{2+}levels. Similarly, we assume that the weights T_{ijkl} between astrocyte processes ij and kl is only a function of indices j and l. We likewise assume that the synapses only receive pre-synaptic input. That is,

$$\tau\ \dot{x}_i = -x_i + \sum_{j=1}^{N} g_{ij}\phi_j + b_i$$

$$\tau\ \dot{s}_{ij} = -s_{ij} + \phi_j + \psi_j$$

$$\tau\ \dot{p}_j = -p_j + \sum_{l=1}^{N} T_{jl}\psi_l + s_j$$

where $g_{ij} = W_{ij}\ \tanh(s_{ij})$, W_{ij} is a trainable parameter, and ψ and ϕ are both also hyperbolic tangent. To match the dimensionality of the Tiny ImageNet dataset, our network contains $N = 12288 = 64 \times 64 \times 3$ neurons. We numerically integrate the network using Euler integration for 20 timesteps, using a step-size of $\mathrm{dt} = 0.1\tau$. We set $\tau = 1$ in our experiment. As described in the main text, we initialized the neurons in the network as the masked images. The synapses and astrocyte processes we initialized at zero. The output of the network was a linear layer followed by a sigmoid function, to ensure valid RGB values. The network was trained using the Adam optimizer with a learning rate of 0.001, using a batch size of 64 images. We trained on a subset of 5000 images in the TinyImage dataset, which enabled our network to learn quickly.

6. Additional Experiments

A. Audio Processing. To further evaluate the robustness of the neuron-astrocyte network, we applied it to audio processing. Specifically, we tested its ability to store and reconstruct speech data. First, we converted raw speech waveforms into spectrograms (S3), downsampled them, and quantized them to four-bit precision, yielding approximately 20,000 bits per spectrogram. Following the methodology described in the main text, we stored these representations in the neuron-astrocyte network.

To assess the network's error-correction capabilities, we introduced corruption into the stored spectrograms. The network successfully mitigated these distortions, restoring the original audio signal (S3). These results demonstrate the adaptability of our framework to auditory data and underscore its potential for broader multimodal applications.

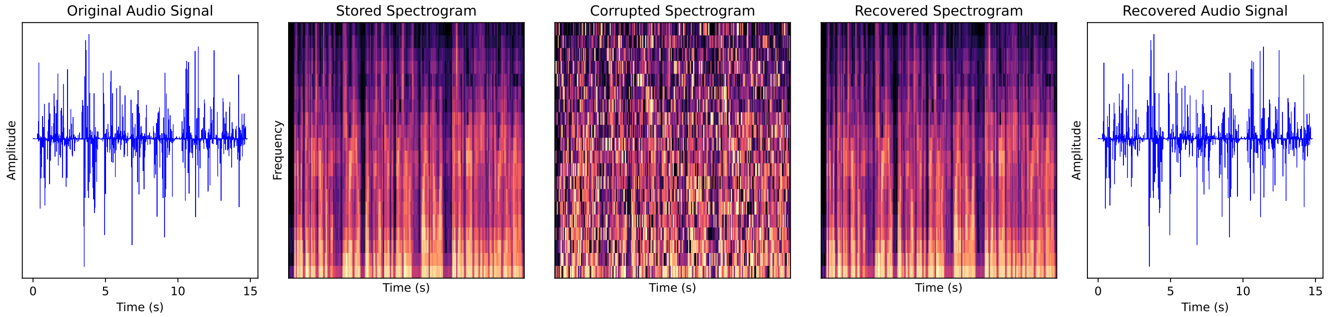

Fig. S3. Reconstructed speech data using neuron-astrocyte networks.

B. Synaptic and Process Patterns at Fixed Points. In this section, we investigate whether distinct patterns emerge in the synaptic weights and process states of our network after training. To explore this, we stored images from the MNIST dataset (handwritten digits) and tested the network's error-correcting capabilities in retrieving them. Once the network converged to a fixed point, we examined the resulting synaptic weight matrix s_{ij} from main text equation (6), and the effective strength of synaptic connections $g_{ij} = \tanh(\beta s_{ij})$. In this specific experiment these two variables are related by a monotone function; for this reason we will plot g_{ij} below. Specifically, we visualized each row of this matrix—where each row contains 784 weights corresponding to the 28×28 pixel dimensions of an image—as a 28×28 grid (S4).

A clear pattern emerged: the synaptic weights at the fixed point formed a mirror image of the recalled memory itself. This phenomenon can be explained through a straightforward theoretical analysis. At the fixed point, the synaptic weight matrix satisfies the relationship:

$$g_{ij} = \sum_{k,l=1}^{N} T_{ijkl}\ \phi_k \phi_l \approx C\ \xi_i^{\hat{\mu}} \xi_j^{\hat{\mu}} + \text{small contributions of sub-leading memories}$$

where $\xi_i^{\hat{\mu}}$ represent the stored pattern that the network converges to and C is a positive constant (this result can be obtained by combining main text equations (8) and (11) in the limit $\alpha = \gamma = 0$). Consequently, plotting the row g_{1j} effectively visualizes the vector $\xi_1^{\hat{\mu}} \xi_i^{\hat{\mu}}$. Since $\xi_1^{\hat{\mu}}$ takes values of ± 1, and $\xi_i^{\hat{\mu}}$ corresponds to the recalled image, the resulting visualization inherently resembles the stored image. The same analysis applies to astrocyte processes, yielding similar results.

Supporting Information References

1. D. Tyulmankov, C. Fang, A. Vadaparty, G. R. Yang, Biological learning in key-value memory networks. *Adv. Neural Inf. Process. Syst.* **34**, 22247–22258 (2021).
2. J. J. Hopfield, Neurons with graded response have collective computational properties like those of two-state neurons. *Proc. Natl. Acad. Sci. U.S.A.* **81**, 3088–3092 (1984).
3. M. A. Cohen, S. Grossberg, Absolute stability of global pattern formation and parallel memory storage by competitive neural networks. *IEEE Trans. Syst. Man Cybern.* **13**, 815–826 (1983).
4. W. Wang, J.-J. E. Slotine, On partial contraction analysis for coupled nonlinear oscillators. *Biol. Cybern.* **92**(1), 38–53 (2005).
5. A. Rahimi, B. Recht, Random features for large-scale kernel machines. *Adv. Neural Inf. Process. Syst.* **20**, 1177–1184 (2007).

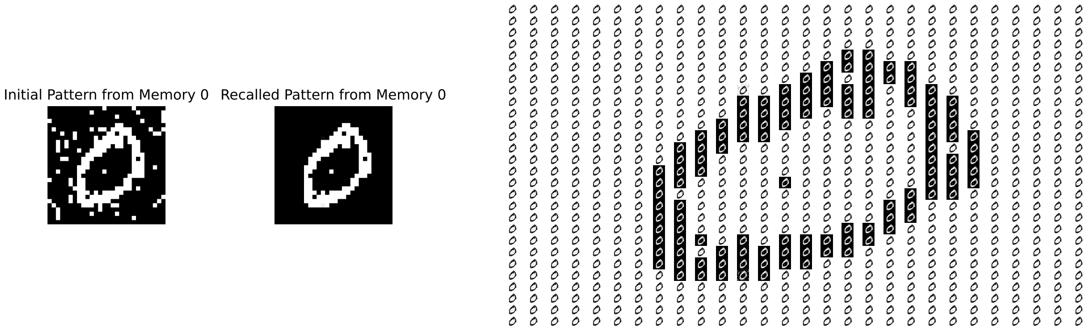

Fig. S4. Synaptic pattern on MNIST example. The leftmost plot shows the initial state of the neuron-astrocyte network. The middle plot shows the state of the network at the fixed point. The rightmost plot shows each row of the synaptic weight matrix g_{ij}, evaluated at the fixed point.
